# Association of sarcopenia with incident osteoporosis: a prospective study of 168,682 UK biobank participants

**DOI:** 10.1002/jcsm.12757

**Published:** 2021-07-15

**Authors:** Fanny Petermann‐Rocha, Lyn D. Ferguson, Stuart R. Gray, Irene Rodríguez‐Gómez, Naveed Sattar, Stefan Siebert, Frederick K. Ho, Jill P. Pell, Carlos Celis‐Morales

**Affiliations:** ^1^ Institute of Health and Wellbeing University of Glasgow Glasgow UK; ^2^ British Heart Foundation Glasgow Cardiovascular Research Centre, Institute of Cardiovascular and Medical Sciences University of Glasgow Glasgow UK; ^3^ GENUD Toledo Research Group, Universidad de Castilla‐La Mancha Toledo Spain; ^4^ CIBER of Frailty and Healthy Aging (CIBERFES), Madrid Spain; ^5^ Institute of Infection, Immunity & Inflammation University of Glasgow Glasgow UK; ^6^ Centre of Exercise Physiology Research (CIFE), Universidad Mayor Santiago Chile; ^7^ Human Performance Lab, Education, Physical Activity and Health Research Unit, University Católica del Maule. Talca 3466706 Chile

**Keywords:** Sarcopenia, Osteoporosis, Muscle strength, Physical capability

## Abstract

**Background:**

Sarcopenia often co‐occurs with osteoporosis in cross‐sectional studies. However, this association has rarely been studied in prospective studies. This study aimed to investigate the association between sarcopenia categories—along with its individual components—and incident osteoporosis in both middle‐aged and older men and women from the UK Biobank study.

**Methods:**

A total of 168,682 participants (48.8% women, aged 37 to 70 years at baseline) were included in this prospective study. Categories of sarcopenia (pre‐sarcopenia and sarcopenia), and its individual components, were defined according to the EWGSOP2 criteria (2019). Associations with incident osteoporosis by sex were investigated using Cox‐proportional hazard models adjusted for socio‐demographic, lifestyle and health‐related factors, and morbidity count. Associations between categories of sarcopenia and incident osteoporosis were also investigated by age‐groups and subtype of osteoporosis (with and without pathological fractures).

**Results:**

After a median follow‐up of 7.4 years, 6296 participants were diagnosed with osteoporosis. When the analyses were adjusted for a range of relevant confounding factors, pre‐sarcopenia was associated with 1.3‐times higher risk of osteoporosis in men (HR: 1.30 [95% CI: 1.03 to 1.63]) but not in women, and sarcopenia was associated with 1.66‐times increased osteoporosis risk in women (HR: 1.66 [95% CI: 1.33 to 2.08]) but not in men compared with people without sarcopenia or pre‐sarcopenia. A similar magnitude of associations was found in osteoporosis without pathological fractures but weaker for those with pathological fractures. Within the individual components, low muscle mass (HR_women_: 1.36 [95% CI: 1.22 to 1.51] and HR_men_: 3.07 [95% CI: 1.68 to 5.59]), followed by slow gait speed (HR_women_: 1.30 [95% CI: 1.17 to 1.45] and HR_men_: 1.70 [95% CI: 1.43 to 2.02]), were associated with a higher risk of incident osteoporosis in both sexes. Low grip strength was associated with a higher risk of incident osteoporosis in men (HR: 1.38 [95% CI: 1.15 to 1.65]), but not in women. No significant interaction between the exposures and incident osteoporosis by age groups were identified.

**Conclusions:**

Our findings demonstrated that pre‐sarcopenic men and sarcopenic women had a higher risk of developing osteoporosis even after adjustment for a large range of potential confounders. Considering that sarcopenia could be prevented, health interventions to improve physical capability may delay or prevent the onset of osteoporosis.

## Introduction

Osteoporosis is the result of changes in bone turnover that reduces bone mineral density (BMD), increase bone fragility, and predispose to fragility fractures along with a higher burden of morbidity and mortality.^1^ Clinically, osteoporosis is identified as a BMD more than 2.5 standard deviations (SD) below the mean value in younger and healthy individuals (a T‐score of <−2.5 SD).^2^, ^3^ According to the International Osteoporosis Foundation, in 2017, approximately 2.8 million people older than 50 years had osteoporosis in the United Kingdom, while fragility fractures, associated with osteoporosis, are the fourth most common chronic disease after ischaemic heart disease, dementia, and lung cancer.^4^ Moreover, the economic burden and healthcare costs linked to osteoporosis are also high. In 2017, the economic cost of the disease in the United Kingdom was £4.5 billion; however, this is projected to rise to £5.9 billion by 2030.^4^ Therefore, it is important to ascertain risk factors for osteoporosis that help us identify high‐risk individuals and develop interventions aimed at prevention or early treatment, in order to reduce the personal and economic burden of osteoporosis.

Although several risk factors have been linked to a higher risk of osteoporosis, the evidence has not been unequivocal.^1^, ^5^, ^6^ Some of the well‐recognized risk factors for osteoporosis include older age, white ethnic background, post‐menopause in women, weight loss, smoking, excessive alcohol intake, vitamin D deficiency (lack of sunlight exposure), inadequate intake of calcium (lower than 1000 mg/day), low protein intake (lower than 0.8 g/kg/body weight) as well as lack of physical activity. Muscle weakness has also been associated with a higher risk of osteoporosis independently of physical activity.^5^, ^6^ In keeping with this finding, the age‐related decline in muscle quantity and quality, known as sarcopenia, also affects mobility, bone mass, and bone microarchitecture. In fact, existing evidence has suggested that sarcopenia may be an independent predictor of low BMD and fragility fractures, that is, osteoporosis.^7^, ^8^, ^9^, ^10^, ^11^, ^12^

Previous studies have reported that sarcopenia and osteoporosis often co‐occur.^7^, ^8^, ^10^, ^11^, ^12^ A recent meta‐analysis identified that the prevalence of osteoporosis and sarcopenia in white European aged 65 years or older varied between 5.0% and 37.0%.^13^ Unfortunately, the cross‐sectional nature of most existing evidence has limitations and does not allow further understanding of the association between sarcopenia and osteoporosis. In terms of prospective evidence, the majority of these studies have investigated the association between individual physical capability markers and sarcopenia with fracture risk^14^, ^15^, ^16^, ^17^, ^18^, ^19^, ^20^, ^21^, ^22^ and have been conducted on smaller samples (*n* < 5000) or focused mainly on older adults. Also, to our knowledge, there are no studies which have investigated the prospective association between sarcopenia and incident osteoporosis *per se*. Therefore, this study aimed to investigate the association between sarcopenia categories—along with its individual components—and incident osteoporosis in both middle‐aged and older men and women, using data from UK Biobank, a large prospective cohort study.

## Methods

Over 500,000 participants (5.5% response rate), aged 37 to 73 years, were recruited from the general population between 2006 and 2010 to be part of UK Biobank.^23^ In brief, participants attended their closest assessment centre across Scotland, England, and Wales^24^, ^25^ where they completed a touch‐screen questionnaire, had physical measurements taken, and provided blood, urine, and saliva sample at baseline. More information about the UK Biobank protocol can be found online (http://www.ukbiobank.ac.uk).

### Incident osteoporosis

Incident osteoporosis cases were ascertained through linkage of primary care records. Diagnosis of osteoporosis was primarily based on dual‐energy X‐ray absorptiometry (DXA) scan results. However, women >75 years that experienced a fragility fracture may be diagnosed with osteoporosis prior to a DXA scan. Currently, this information was available only for 45% of the UK Biobank cohort (~230,000 participants) until May 2017 for Scotland, September 2017 for Wales, and August 2017 for England. The detailed linkage procedures relating to primary care records are available at http://biobank.ndph.ox.ac.uk/showcase/showcase/docs/primary_care_data.pdf. Therefore, the analyses of incident osteoporosis cases were restricted to the 228,481 participants with linkage to primary care records. Follow‐up was censored at the primary care data end‐date for the relevant country or the date of incident osteoporosis. Osteoporosis was defined as M80 (osteoporosis with pathological fracture) M81 (osteoporosis without pathological fracture) or M82 (osteoporosis in diseases classified elsewhere) using the International Classification of Diseases, 10th revision (ICD‐10).

### Sarcopenia and its components

Muscle mass index was derived from skeletal muscle mass (kg) divided by height (m) squared using the total body composition measured via bioimpedance (BIA, Tanita BC418MA, Tokyo, Japan) by trained nurses. To estimate skeletal muscle mass, the Janssen equation was utilized.^26^ Following the European Working Sarcopenia in Older People 2019 (EWGSOP2) recommendations, the cut‐off points used were <7.0 kg/m^2^ in men and <5.5 kg/m^2^ in women. Grip strength was measured using a Jamar J00105 hydraulic hand dynamometer. The mean of the right and left values was derived and expressed in absolute units (kg). The cut‐off points applied to define low grip strength were <27 kg in men and <16 kg in women.^27^ Self‐reported walking pace was used as a proxy of gait speed and categorized as slow, average or brisk. A previous study determined that self‐reported walking pace is a good marker of walking speed.^28^ To derive a proxy for gait speed, this was then dichotomized into slow or normal (average or brisk pace).

Using these three physical capability markers, sarcopenia was classified in accordance with the EWGSOP2 statement as pre‐sarcopenia, defined as low grip strength only (other physical capability markers in the normal range); sarcopenia, defined as low grip strength plus low muscle mass^27^; and severe sarcopenia, defined as the combination of sarcopenia and slow gait speed. However, because of the low number of UK Biobank participants with severe sarcopenia (*n* = 87), sarcopenia and severe sarcopenia were pooled together (hereafter referred to as sarcopenia). We followed this approach to avoid unreliable and unpowered hazard ratios (HR) estimates. The pre‐sarcopenia and sarcopenia groups were mutually exclusive. For this study, only white European participants were included because of the ethnic differences in the reference values for sarcopenia.^27^

### Covariates

Age at baseline was calculated from dates of birth and baseline assessment. Area‐based socioeconomic deprivation was derived from postcode of residence, using the Townsend score.^29^ Self‐reported smoking status was categorized as never, former or current smoker. Physical activity was self‐reported using the International Physical Activity Questionnaire short form^30^ and total physical activity was computed as the sum of walking, moderate and vigorous activity, measured as metabolic equivalents (MET‐hours/week). Prevalent morbidity was ascertained during a nurse‐led interview at baseline. We calculated morbidity count based on 43 long‐term conditions originally developed for a large epidemiological study in Scotland and subsequently adapted for UK Biobank.^31^ Body composition was measured using BIA by trained nurses. Frequency of alcohol intake was self‐reported at baseline and categorized as daily/almost daily, three to four times a week, once/twice a week, one to three times a month, special occasions only and never. Corticosteroid and H2 blockers use, as well as menopause and hypogonadism, were self‐reported at baseline. History of fall and fractures were self‐reported at baseline using these two questions: ‘In the last year, have you had any falls?’ and ‘have you fractured/broken any bones in the last five years?’ Vitamin D levels were assessed by 25‐hydroxyvitamin D (25(OH)D) concentration in serum. Red and processed meat intake were collected through the touch‐screen questionnaire at baseline. Finally, calcium and protein intake were estimated via the Oxford WebQ, a web‐based 24‐h recall questionnaire.^32^ For the 71,673 participants who completed more than one the average dietary of the 24‐h recall, this average intake was used. Further details of these measurements can be found in the UK Biobank online protocol (http://www.ukbiobank.ac.uk).

### Ethical approval

UK Biobank was given favourable opinion by the North West Multi‐Centre Research Ethics Committee (Ref: 11/NW/0382). The study protocol is available online (http://www.ukbiobank.ac.uk/). This work was conducted under the UK Biobank application number 7155.

### Statistical analyses

Descriptive characteristics are presented as means with standard deviations (SD) for quantitative variables, and as frequencies and percentages for categorical variables by sex.

Associations between categories of sarcopenia (pre‐sarcopenia and sarcopenia) and incident osteoporosis were investigated using Cox‐proportional hazard models stratified by sex. Non‐sarcopenic individuals (i.e. with the three physical capability markers in the normal range) were used as the reference group. The results are reported as HR and their 95% confidence intervals (95% CIs). The proportional hazard assumptions were checked using Schoenfeld residuals. Associations between the three individual physical capability markers (low grip strength, low muscle mass and slow gait speed) and incident osteoporosis were investigated using the same analysis. For each component, the normal range of the physical capability maker defined by the EWGSOP2 was used as the reference group.^27^ Participants who self‐reported osteoporosis at baseline were excluded from all analyses (*n* = 3472). Only participants with complete data available for the three physical capability markers used to define later categories of sarcopenia, the covariates included in the analyses, and incident osteoporosis were included. Follow‐up time was used as the time‐dependent variable.

We ran five models including an increasing number of covariates: model 1 (minimally adjusted) included socio‐demographic covariates (age and deprivation); model 2 additionally included lifestyle and health‐related factors: smoking, physical activity, alcohol intake and consumption of red and processed meat, body fat, and morbidity count at baseline (based on 43 diseases and coded as 1, 2, 3, 4 and ≥5). Model 3, as model 2, but additionally adjusted for serum vitamin D levels, use of corticosteroids and H2 blockers, falls, fractures over the previous 5 years, and menopause and hypogonadism in women and men, respectively. These covariates were chosen as they are potentially causal for sarcopenia and osteoporosis. Two sensitivity analyses were also performed: model 4, as per model 3, but using a 2‐year landmark period which excluded participants who experienced events within the first 2 years of follow‐up (1323 women and 290 men) to minimize potential reverse causation; and model 5, as per model 3, but further adjusted for calcium and protein intake (as these variables were available in 71,673 participants only). Additionally, the association of categories of sarcopenia with subtypes of osteoporosis outcomes (split out by pathological fractures and osteoporosis without pathological fractures [or classified elsewhere]) was also investigated.

The sex‐specific cumulative crude hazard rate of incident osteoporosis and categories of sarcopenia was estimated using the Nelson–Aalen estimator. Finally, to investigate whether the associations between categories of sarcopenia and incident osteoporosis differed by age, the models were re‐run stratified by the following age categories: (i) approximately when menopause or hypogonadism start (≥45 and <45 years as well as ≥55 and <55 years), (ii) using different definitions for aging (≥60 and <60 years as well as ≥65 and <65 years).

Stata 16 statistical software (StataCorp LP) was used to perform all analyses.

## Results

**Figure 1:**
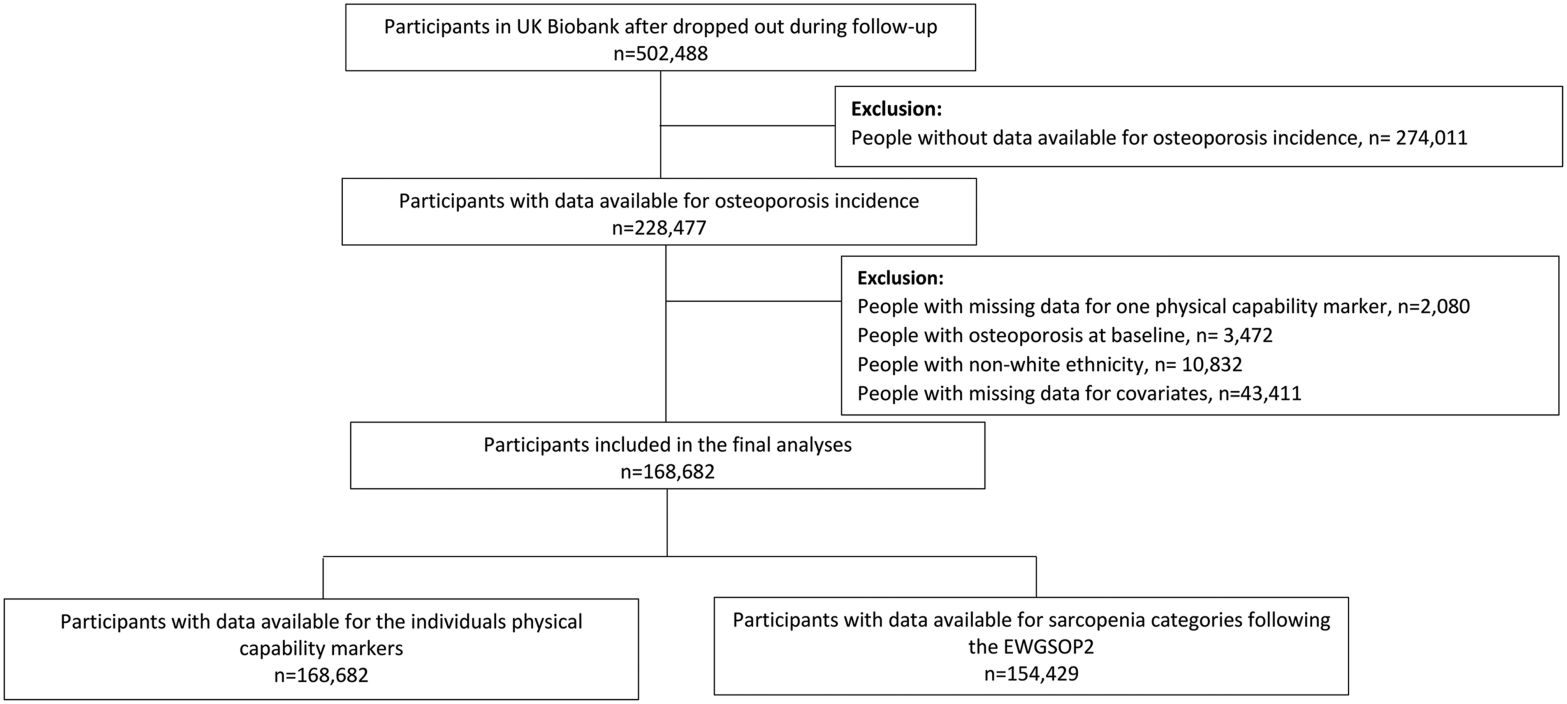
Flow diagram participants included in the study. EWGSOP2, European working sarcopenia in older people 2019.

After removing people who withdrew during the follow‐up, 228,477 of the 502,488 UK Biobank participants had data available for incident osteoporosis. Excluding people with missing data for one or more physical capability marker (*n* = 2080), osteoporosis at baseline (*n* = 3472), non‐white ethnicity (*n* = 10,832) or incomplete covariate data (*n* = 43,411), 168,682 participants (48.8% women) had data available on all essential variables (*Figure* 1). Of these, 154,429 could be classified as non‐sarcopenia, pre‐sarcopenia, or sarcopenia (Supporting Information, *Figure* S1). After a median follow‐up of 7.4 years (interquartile range 6.7 to 8.2 years), 6296 (3.7%) participants were diagnosed with osteoporosis.

The baseline characteristics of participants by sarcopenia categories and sex are shown in *Table* 1. Briefly, 5950 (8.0%) of the 74,293 women and 4075 (5.1%) of the 80,136 men were pre‐sarcopenic or sarcopenic. Overall, compared with non‐sarcopenic individuals, both men and women with pre‐sarcopenia or sarcopenia were older, more likely to currently smoke, use H2 blockers and/or corticosteroids, and report never drinking alcohol. They had lower levels of physical activity and reported a lower intake of protein and calcium. They were also more likely to have more than one morbidity, and to have had fractures in the last 5 years and falls in the last year. Lastly, pre‐sarcopenic and sarcopenic individuals were more likely to be postmenopausal (women) and report hypogonadism (men) compared with non‐sarcopenic women and men, respectively (*Table* 1). The baseline characteristics by individual physical capability marker by sex are shown in *Tables* S1 to S3.

**Table 1:** Baseline characteristics by categories of sarcopenia and sex

	Total	Women			Men		
		Non‐sarcopenic	Pre‐sarcopenia*	Sarcopenia*	Non‐sarcopenic	Pre‐sarcopenia*	Sarcopenia*
**Socio‐demographics**							
Total, *n* (%)	154,429 (100)	68,343 (92.0)	5430 (7.3)	520 (0.7)	76,061 (94.9)	4036 (5.0)	39 (0.1)
Age (years), mean (SD)	56.2 (8.1)	55.4 (8.1)	59.9 (7.0)	62.5 (5.4)	56.5 (8.1)	59.9 (7.4)	60.9 (7.2)
Deprivation, *n* (%)							
Lower	54,620 (35.4)	24,234 (35.5)	1590 (29.3)	153 (29.4)	27,539 (36.2)	1094 (27.1)	10 (25.6)
Middle	53,924 (34.9)	24,019 (35.1)	1968 (36.2)	210 (40.4)	26,377 (34.7)	1341 (33.2)	9 (23.1)
Higher	45,885 (29.7)	20,090 (29.4)	1872 (34.5)	157 (30.2)	22,145 (29.1)	1601 (39.7)	20 (51.3)
**Lifestyle**							
Body fat (kg), mean (SD)	24.1 (8.8)	26.2 (9.4)	26.9 (9.3)	22.4 (6.4)	22.0 (7.8)	22.5 (8.1)	19.2 (8.4)
Total PA (MET/h/week), mean (SD)	3067.3 (3345.0)	2751.3 (2839.4)	2707.0 (2852.2)	2562.4 (2741.2)	3378.1 (3732.8)	2998.0 (3503.0)	1938.4 (1675.7)
Total sedentary behaviour (h/day), mean (SD)	5.0 (2.2)	4.6 (1.9)	4.7 (2.0)	4.6 (1.9)	5.4 (2.4)	5.4 (2.5)	4.7 (2.9)
Red meat (portion.week^‐1^), mean (SD)	2.1 (1.4)	2.0 (1.3)	2.0 (1.3)	1.9 (1.3)	2.2 (1.4)	2.3 (1.6)	2.2 (1.6)
Processed meat intake (portion.week^‐1^), mean (SD)	1.9 (1.1)	1.6 (1.0)	1.6 (1.0)	1.7 (1.0)	2.2 (1.0)	2.2 (1.1)	2.0 (1.2)
Protein (g/day), mean (SD)	83.3 (25.8)	78.7 (23.1)	78.1 (23.8)	74.2 (22.7)	87.8 (27.5)	85.2 (26.7)	81.6 (22.4)
Calcium (mg/day), mean (SD)	995.4 (389.4)	959.5 (370.3)	960.8 (390.2)	877.3 (356.2)	1031.2 (403.9)	998.7 (373.5)	960.6 (292.7)
Alcohol frequency intake, *n* (%)							
Daily or almost daily	3868 (21.3)	11,403 (16.7)	805 (14.8)	91 (17.5)	19,560 (25.7)	994 (24.6)	1298 (20.9)
3–4 times a week	38,929 (25.2)	15,478 (22.7)	974 (17.9)	85 (16.4)	21,415 (28.2)	970 (24.0)	1177 (18.9)
Once or twice a week	42,235 (27.4)	19,007 (27.8)	1509 (27.8)	129 (24.8)	20,512 (27.0)	1072 (26.6)	1618 (26.0)
1–3 times a month	17,014 (11.0)	9212 (13.5)	655 (12.1)	64 (12.3)	6718 (8.8)	362 (9.0)	608 (9.7)
Special occasions only	14,595 (9.4)	8705 (12.7)	913 (16.8)	88 (16.9)	4565 (6.0)	322 (8.0)	782 (12.6)
Never	8788 (5.7)	4538 (6.6)	574 (10.6)	63 (12.1)	3291 (4.3)	316 (7.8)	740 (11.9)
Smoking status, *n* (%)							
Never	84,809 (54.9)	41,026 (60.0)	3177 (58.5)	295 (56.7)	38,319 (50.4)	1972 (48.9)	20 (51.3)
Previous	54,546 (35.3)	21,663 (31.7)	1832 (33.7)	176 (33.9)	29,264 (38.5)	1600 (39.6)	11 (28.2)
Current	15,074 (9.8)	5654 (8.3)	421 (7.8)	49 (9.4)	8478 (11.1)	464 (11.5)	8 (20.5)
Health status							
Multimorbidity, *n* (%)							
0	57,877 (37.5)	27,058 (39.6)	1348 (24.8)	111 (21.4)	28,336 (37.3)	1012 (25.1)	12 (30.8)
≥1	96,552 (62.5)	41,285 (60.4)	4082 (72.2)	409 (78.6)	47,725 (62.7)	3024 (74.9)	27 (69.2)
Vitamin D (nmol/L), mean (SD)	49.2 (20.8)	48.8 (20.6)	49.3 (20.4)	50.4 (22.0)	49.6 (21.0)	48.7 (20.8)	40.6 (25.7)
Using H2 blockers, *n* (%)	2558 (1.7)	1049 (1.5)	135 (2.5)	15 (2.9)	1265 (1.7)	93 (2.3)	1 (2.6)
Using steroid, *n* (%)	1420 (0.9)	532 (0.8)	79 (1.4)	12 (2.3)	722 (1.0)	74 (1.8)	1 (2.6)
Fractures in the last 5 years, *n* (%)	13,317 (8.7)	6059 (8.9)	645 (11.9)	70 (13.5)	6163 (8.2)	373 (9.3)	7 (18.0)
Falls in the last year, *n* (%)							
No falls	128,081 (83.0)	54,785 (80.3)	3901 (72.0)	373 (71.9)	65,813 (86.6)	3189 (79.3)	20 (63.9)
Only one fall	18,835 (12.2)	9978 (14.6)	987 (18.2)	105 (20.2)	7200 (9.5)	553 (13.7)	866 (14.0)
More than one fall	7322 (4.8)	3512 (5.1)	528 (9.8)	41 (7.9)	2952 (3.9)	282 (7.0)	1368 (22.1)
Hypogonadism, *n* (%)	356 (0.2)	‐	‐	‐	328 (0.4)	28 (0.7)	39 (100)
Menopause, *n* (%)	51,366 (69.1)	46,110 (67.5)	4753 (87.5)	503 (96.7)	‐	‐	‐

MET, metabolic‐equivalent; *n*, number; PA, physical activity; SD, standard deviation; ‐, no data available.

* Sarcopenia includes those with sarcopenia or severe sarcopenia.

**Figure 2:**
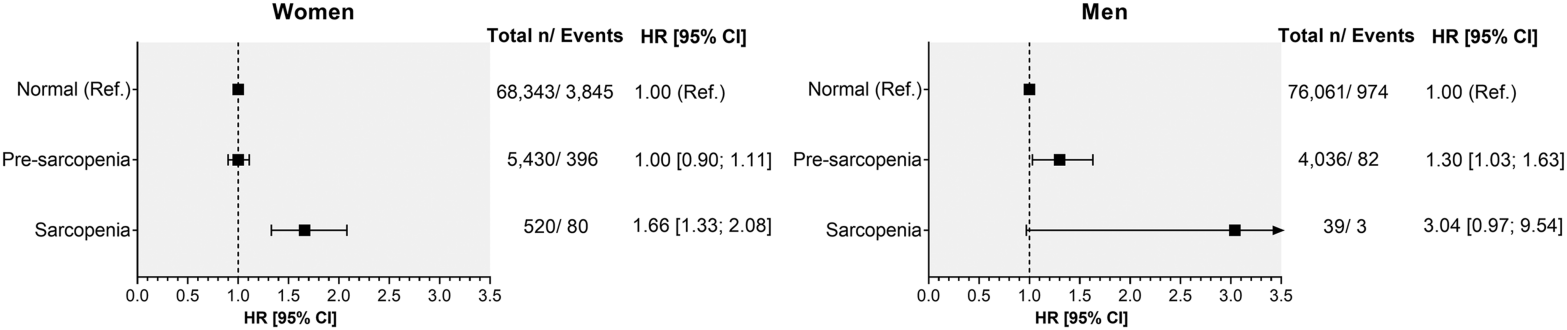
Association between categories of sarcopenia and osteoporosis incidence by sex. Analyses are presented as HR with their respectively CI. Non‐sarcopenic participants were used as the reference group. Analyses were adjusted by socio‐demographic factors (age and deprivation), morbidity count, physical activity, smoking, alcohol and red and processed meat intake, body fat, serum vitamin D levels, corticosteroids, H2 blockers, falls and fractures in the last 5 years and menopause in women and hypogonadism in men (model 3). *Sarcopenia includes those with sarcopenia or severe sarcopenia.

Sex‐specific associations between sarcopenia categories and incident osteoporosis are shown in *Figure* 2 and *Table* S4. In the minimally adjusted model (model 1), a higher risk of incident osteoporosis was identified in sarcopenic women compared to non‐sarcopenic (HR: 2.01 [95% CI: 1.61 to 2.51]). The association was attenuated after adjustment for lifestyle factors, body composition and morbidity count (model 2) and remained significant in model 3 (HR: 1.66 [95% CI: 1.33 to 2.08]). The results were similar in the 2‐year landmark analysis (model 4). However, the association were no longer present when protein and calcium intake were included as covariates (model 5). No associations were identified between pre‐sarcopenic women and incident osteoporosis. Incident osteoporosis without pathological fractures showed similar patterns of associations (*Table* S5). Additionally, pre‐sarcopenic women showed a higher risk of incident osteoporosis using this outcome (HR_model 3:_ 1.15 [95% CI: 1.01 to 1.32]). Non‐significant associations were identified between categories of sarcopenia and osteoporosis with pathological fractures.

The risk of osteoporosis in pre‐sarcopenic and sarcopenic men was 1.40‐ and 4.97‐times higher, respectively, in comparison to non‐sarcopenic men in the minimally adjusted model (model 1). The associations were attenuated when the analysis was further adjusted for morbidity count, lifestyle and health‐related factors (models 2 and 3) for sarcopenic men but remained significant for pre‐sarcopenic men (HR: 1.30 [95% CI: 1.03 to 1.63]) (*Figure* 2). As per women, a similar trend was identified when a two‐year landmark was included in the analysis but disappeared when protein and calcium intake were included (model 5, *Table* S4). When the subtypes of osteoporosis were used as outcomes, we observed a similar magnitude of association between pre‐sarcopenic men and osteoporosis without pathological fractures. The associations with the pathological fracture incidence were non‐significant (*Table* S5). On the other hand, regarding the cumulative hazard estimate, both men and women with sarcopenia had a steeper crude cumulative incidence of osteoporosis than non‐sarcopenic men and women, respectively (*Figures* S2 and S3).

**Figure 3:**
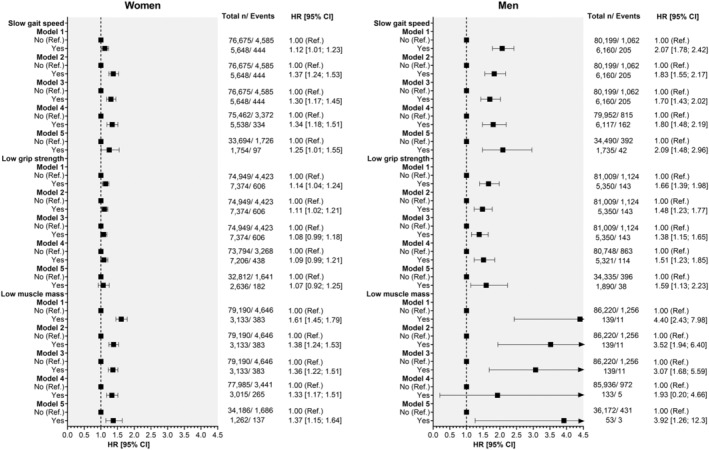
Associations between individual physical capability markers and incident osteoporosis by sex. Analyses are presented as HR with their respectively CI. Non‐sarcopenic participants were used as the reference group. Analyses were adjusted by model 1, adjusted by socio‐demographic factors (age and deprivation); model 2 as model 1, but additionally morbidity count, physical activity, smoking, body fat, alcohol and red and processed meat intake. Model 3, as model 2, but additionally adjusted by serum vitamin D levels, corticosteroids, H2 blockers, falls and fractures in the last 5 years and menopause in women and hypogonadism in men. Model 4, as per model 3, but using a 2‐year landmark that excluded participants who experienced events within the first 2 years of follow‐up; and model 5, as per model 3, but further adjusted for calcium and protein intake.

Of the three physical capability markers used to define categories of sarcopenia, slow gait speed and low muscle mass were independently associated with 1.30‐ and 1.36‐times higher risk of incident osteoporosis in women and 1.70‐ and 3.07‐times higher risk of incident osteoporosis in men, respectively (*Figure* 3, model 3). The associations remained when the analyses were further adjusted for protein and calcium intake and in the two‐year landmark analysis (except for low muscle mass in men, probably due to the few numbers of cases). Low grip strength was associated with a higher risk of incident osteoporosis in men across all models (HR_model 3_:1.38 [95% CI: 1.15 to 1.65]), but not in women. Based on model 3, low muscle mass was the physical capability marker associated with the highest risk of incident osteoporosis in both sexes.

Finally, while there were no significant interactions with age‐group, the numerical magnitude of the associations between sarcopenia and incident osteoporosis was higher in the older age‐group for sarcopenic women and pre‐sarcopenic men compared to their counterparts (*Table* S6).

## Discussion

Sarcopenia and osteoporosis are prevalent conditions that are associated with substantial health burden.^1^ After adjustment for a wide range of potential confounding factors, pre‐sarcopenia was associated with a higher risk of incident osteoporosis in men, but not in women, while sarcopenia was associated with a higher risk in women, but not in men. The lack of association between sarcopenic men and incident osteoporosis might be related to the low number of sarcopenic men in our study; therefore, this analysis was probably underpowered. These results were consistent for individuals without pathological fractures but not for those with osteoporosis with pathological fractures. The latter reinforces the relevance of the early assessment of sarcopenia in these individuals beyond fractures. Among the three physical capability markers used to define sarcopenia, low muscle mass was associated with the highest risk of incident osteoporosis in both sexes, followed by slow gait speed (in both sexes) and low grip strength (in men only). Given these findings, and considering the health and economic burden of osteoporosis in the United Kingdom,^4^ preventing, diagnosing and treating sarcopenia might help prevent or delay some cases of osteoporosis and the significant health and financial burden associated with this, assuming causality. As the decrease in muscle mass starts at ~40 years,^27^ and this leads to a higher risk of falls and fragility fractures, interventions improving or maintaining decent physical capability levels in middle and older ages are needed.

The association between sarcopenia and osteoporosis has been previously studied, but most evidence comes from cross‐sectional studies.^7^, ^8^, ^10^, ^11^, ^12^ To our knowledge, this is the first study reporting the longitudinal association between sarcopenia and incident osteoporosis. Previous prospective studies often used fractures as a proxy for osteoporosis, even though all fractures may not necessarily indicate osteoporosis and, as it was demonstrated in our study, pre‐sarcopenic men and sarcopenic women had a higher risk of osteoporosis without pathological fractures. In terms of fracture studies, Yu et al. used the MrOs study to report that sarcopenia was an independent risk factor for fractures in men (HR: 1.87 [95% CI: 1.30 to 2.68]), but not in women (HR: 0.80 [95% CI: 0.49 to 1.31]).^19^ Similarly, Scott et al. identified that sarcopenic obese community‐dwelling older men had more than 3‐times higher rate of self‐reported fractures compared to non‐sarcopenic non‐obese men. Sarcopenic obese women, in contrast, had a higher risk of fracture compared with obese women (incident rate ratio: 2.82 [95% CI: 1.42 to 5.60]), but this was mediated by BMD (incident rate ratio: 1.93 [95% CI: 0.94 to 3.98]).^20^ In comparison to our study, these studies used different classifications to define sarcopenia, their outcome was the risk of fracture instead of osteoporosis itself, and included only older individuals. The latter reinforces the relevance of our findings which identified an increased risk of osteoporosis in both sexes (women with sarcopenia and men with pre‐sarcopenia) using the latest guidelines suggested for the EWGSOP2,^27^ in both middle‐aged and older adults.

In terms of the individual components used to define sarcopenia, the majority of previous studies have investigated their association with risk fractures as an outcome.^14^, ^16^, ^17^ Only a few studies have also reported the association of these individual factors with osteoporosis or fragility fractures associated with osteoporosis.^15^, ^33^, ^34^ Cheung et al., using a subset of 1702 participants from the prospective Hong Kong Osteoporosis study, found that grip strength was strongly associated with fragility fractures and osteoporosis at the hip.^33^ Likewise, for each standard deviation lower in gait speed, there was a 2.16‐times higher risk of hip fractures and 1.33‐times higher risk of major osteoporotic fractures among 351 post‐menopausal women who were followed up for 10 years.^34^ In Canada, after 6 years follow‐up of 9622 men and women older than 40 years, Leslie et al. identified that a decrease in total body lean mass was independently associated with an increased risk of osteoporotic fractures.^15^

As a prospective study of osteoporosis, rather than a proxy, the current study fills gaps in the existing evidence base. However, several challenges remain. The lack of a single classification and definition for sarcopenia remains one of the greatest problems for research into sarcopenia, extending beyond studies of the association between sarcopenia and osteoporosis. Achieving a consensual definition would facilitate the comparison of results across studies that use a common definition and would help translation of the findings into clinical practice. Finally, future prospective studies should investigate the joint association of sarcopenia and osteoporosis, i.e., ‘osteosarcopenia’, on adverse health outcomes. Binkley & Buehring were the first to introduce the concept in 2009 as a subset of older adults with both osteoporosis and sarcopenia.^35^ Although more studies have been carried out since that moment,^36^, ^37^ literature using prospective studies is lacking.^38^

### Strength and limitations

UK Biobank provided the opportunity to test our hypothesis in a large and well characterized general population‐based cohort of middle‐aged and older adults. Consequently, analyses could be adjusted for multiple potential confounders. Moreover, incident osteoporosis was ascertained through linkage primary care records. However, UK Biobank is not representative of the UK population in terms of socio‐demographic, lifestyle and prevalent disease. Therefore, while risk estimates can be generalized,^39^ summary statistics such as the prevalence or incidence of health conditions should not.^40^ Muscle mass was measured using BIA. While this method is not the gold standard, muscle mass estimated using BIA has been shown to have good agreement with DXA (*r* = 0.868).^41^ In addition, owing to insufficient statistical power, we were unable to study severe sarcopenia as a separate category and therefore, we combined sarcopenia and severe sarcopenia. Even so, the number of participants in some sarcopenia categories, especially men, was low, which likely explains the lack of significant association for sarcopenic men. Another potential limitation is the self‐reported gait speed. Although we used self‐reported walking pace as a proxy of gait speed, previous studies have shown that this simple and cheap marker of physical capability has a strong predictive ability for chronic diseases and mortality, even beyond mean fracture risk.^42^, ^43^ In addition, even though our analyses adjusted for a large list of confounding factors, some of the associations identified might be due to residual or unmeasured confounding. Finally, the observational nature of the study does not allow us to infer causality from the association; therefore, future trials should investigate the potential causal link of sarcopenia and physical capability markers with osteoporosis.

In conclusion, our findings demonstrated that pre‐sarcopenic men and sarcopenic women had a higher risk of incident osteoporosis even after adjustment for a large range of potential confounders. Since sarcopenia could be prevented, early public health strategies aimed at improving physical capability may help to prevent or delay some cases of osteoporosis. Randomized trials would help address this question.

## Conflict of interest

None declared.

## Funding

UK Biobank was established by the Wellcome Trust medical charity, Medical Research Council, Department of Health, Scottish Government and the Northwest Regional Development Agency. It has also had funding from the Welsh Assembly Government and the British Heart Foundation. FP‐R receives financial support from the Chilean Government for her PhD (ANID‐Becas Chile 2018 – 72190067).

## Author contribution

F.P.‐R., J.P.P., F.K.H., and C.C.‐M. contributed to the conception and design of the study, advised on all statistical aspects, and interpreted the data. F.P.‐R. performed the literature search. F.P.‐R. performed the analyses with support from J.P.P, F.K.H., and C.C.‐M. All authors critically reviewed this and previous drafts. All authors approved the final draft for submission, with final responsibility for publication. J.P.P, F.K.H., and C.C.‐M. contributed equally to this work and are joint senior authors. F.P.‐R., F.K.H., J.P.P., and C.C.‐M. are the guarantor.

## Copyright

The Corresponding Author has the right to grant on behalf of all authors and does grant on behalf of all authors, a worldwide licence to the Publishers and its licensees in perpetuity, in all forms, formats and media (whether known now or created in the future), to (i) publish, reproduce, distribute, display and store the Contribution, (ii) translate the Contribution into other languages, create adaptations, reprints, include within collections and create summaries, extracts and/or, abstracts of the Contribution, (iii) create any other derivative work(s) based on the Contribution, (iv) to exploit all subsidiary rights in the Contribution, (v) the inclusion of electronic links from the Contribution to third party material wherever it may be located; and, (vi) licence any third party to do any or all of the above.

## Transparency

The lead author, (the manuscript's guarantor), affirms that the manuscript is an honest, accurate, and transparent account of the study being reported; that no important aspects of the study have been omitted; and that any discrepancies from the study as planned (and, if relevant, registered) have been explained.

## Supporting information


**Figure S1.** Diagram – Participants according to the different classification by idividuals capability markers and categories of sarcopenia by sex.
**Figure S2.** Cumulative hazard plot of osteoporosis incidence by categories of sarcopenia and follow‐up time in women.
**Figure S3.** Cumulative hazard plot of osteoporosis incidence by categories of sarcopenia and follow‐up time in men.
**Table S1.** Baseline characteristics by categories of gait speed and sex.
**Table S2.** Baseline characteristics by categories of grip strength and sex.
**Table S3.** Baseline characteristics by categories of muscle mass and sex.
**Table S4.** Associations between categories of sarcopenia with incident osteoporosis by sex.
**Table S5.** Associations between categories of sarcopenia with subtypes osteoporosis incidence by sex
**Table S6.** Associations between categories of sarcopenia and incident osteoporosis by age groups and sex.

## Data Availability

All UK Biobank information is available online on the webpage www.ukbiobank.co.uk. Data access is available through applications. This research was conducted using the application number 7155.
