# Supplementary material for: Association of sarcopenia with incident osteoporosis: a prospective study of 168,682 UK biobank participants
Source: J Cachexia Sarcopenia Muscle. 2021 Jul 15;12(5):1179–88. doi: 10.1002/jcsm.12757 (PMC8517357; doi:10.1002/jcsm.12757)
Supplement: Supplementary file 1 — Figure S1. Diagram – Participants according to the different classification by idividuals capability markers and categories of sarcopenia by sex. Figure S2. Cumulative hazard plot of osteoporosis incidence by categories of sarcopenia and follow‐up time in women. Figure S3. Cumulative hazard plot of osteoporosis incidence by categories of sarcopenia and follow‐up time in men. Table S1. Baseline characteristics by categories of gait speed and sex. Table S2. Baseline characteristics by categories of grip strength and sex. Table S3. Baseline characteristics by categories of muscle mass and sex. Table S4. Associations between categories of sarcopenia with incident osteoporosis by sex. Table S5. Associations between categories of sarcopenia with subtypes osteoporosis incidence by sex Table S6. Associations between categories of sarcopenia and incident osteoporosis by age groups and sex. [file JCSM-12-1179-s001.docx]

**
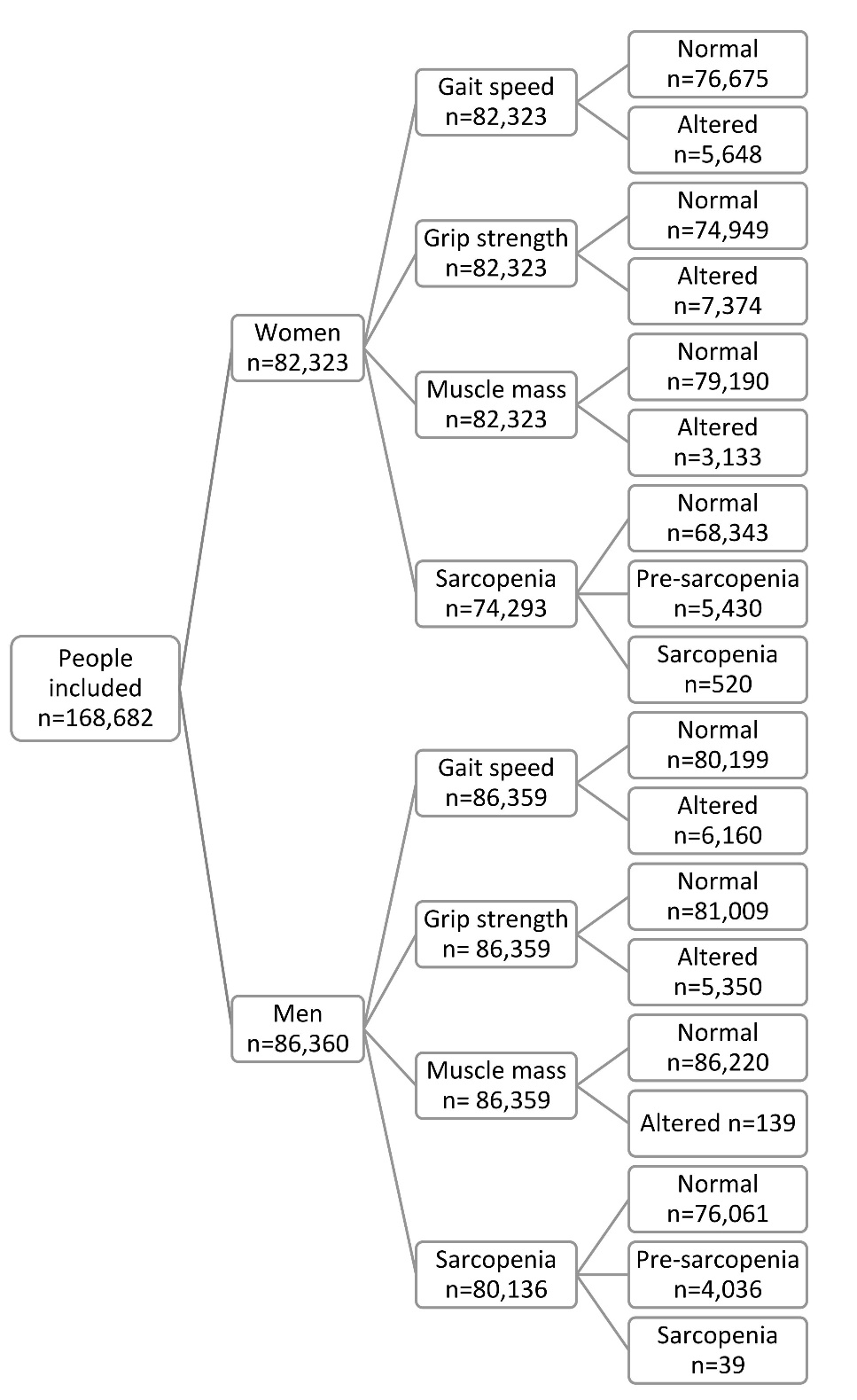
**

**Figure S1. Diagram – Participants according to the different classification by idividuals capability markers and categories of sarcopenia by sex.**

*Sarcopenia includes those with sarcopenia or severe sarcopenia.

**
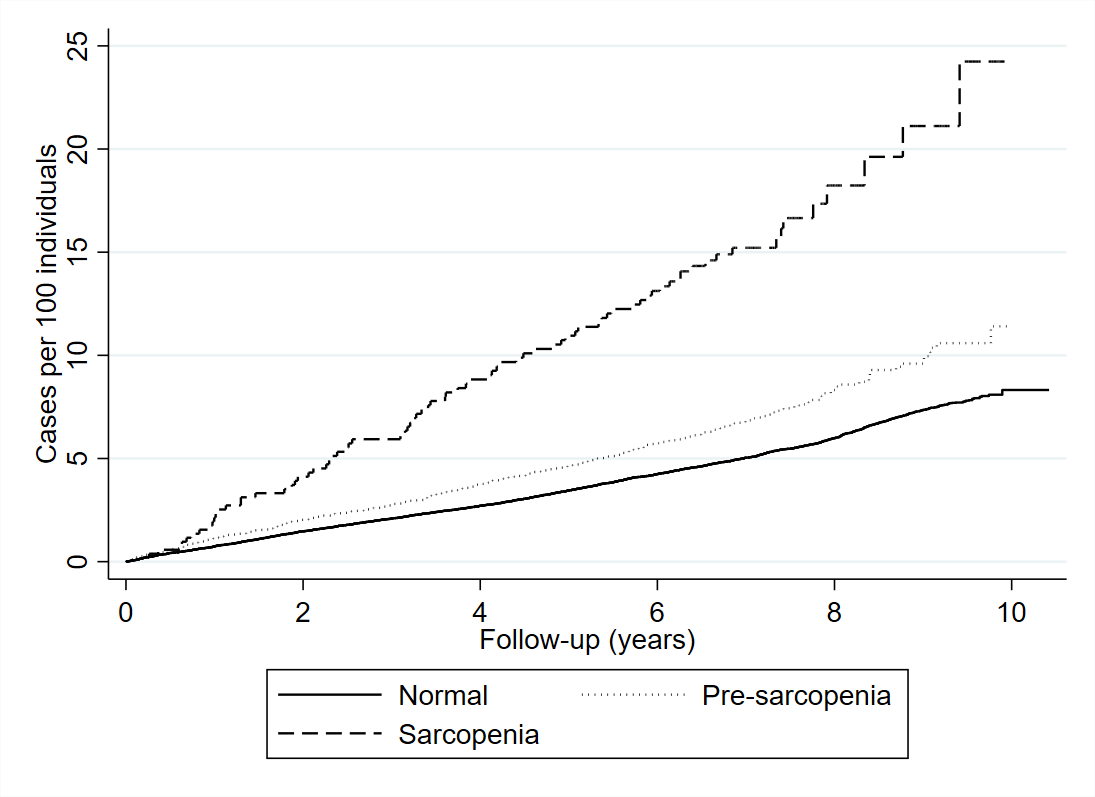
**

**Figure S2. Cumulative hazard plot of osteoporosis incidence by categories of sarcopenia and follow-up time in women.**

Data presented as crude HR by categories of sarcopenia.

**
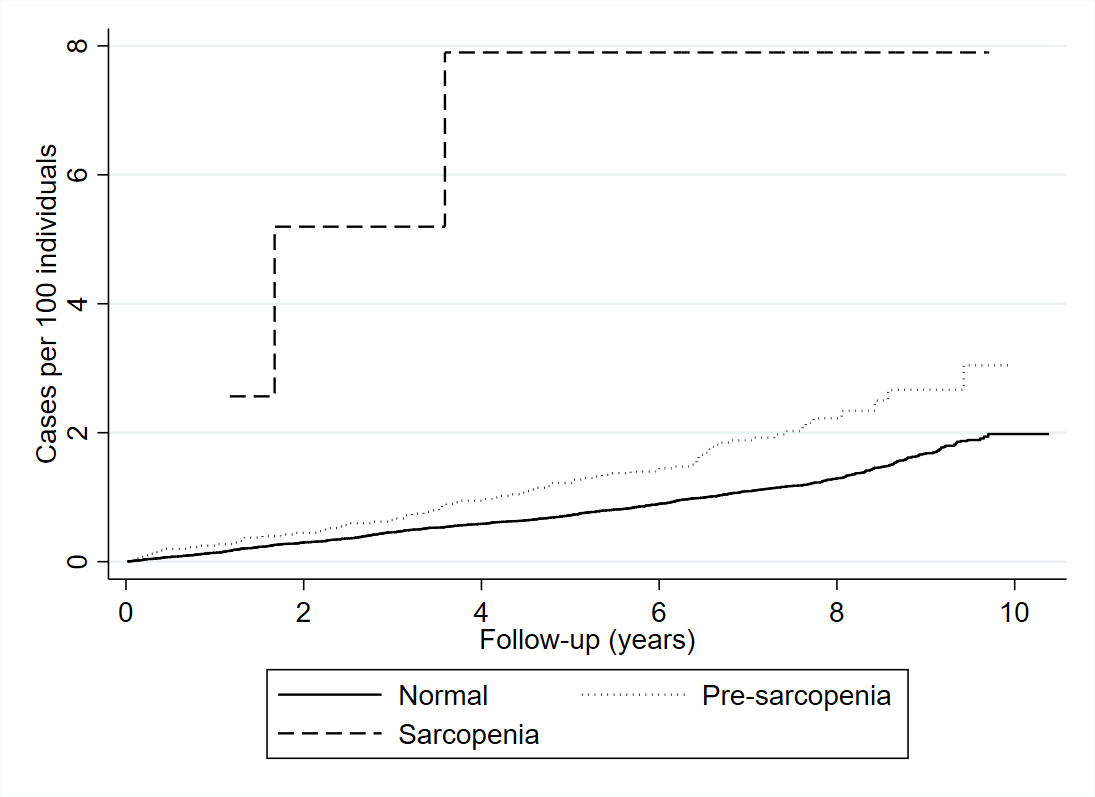
**

**Figure S3. Cumulative hazard plot of osteoporosis incidence by categories of sarcopenia and follow-up time in men.**

Data presented as crude HR by categories of sarcopenia.

**Table S1.** **Baseline characteristics by categories of gait speed and sex**

|  | **Women** | | **Men** | |
| --- | --- | --- | --- | --- |
|  | Normal range | Slow gait speed | Normal range | Slow gait speed |
| **Socio-demographics** |  |  |  |  |
| Total n, (%) | 76,675 (93.1) | 5,648 (6.9) | 80,199 (92.9) | 6,160 (7.1) |
| Age (years), mean (SD) | 55.9 (8.1) | 58.6 (7.5) | 56.7 (8.1) | 60.1 (6.9) |
| Deprivation, n (%) |  |  |  |  |
| Lower | 26,890 (35.1) | 1,344 (23.8) | 28,668 (35.8) | 1,359 (22.1) |
| Middle | 27,031 (35.2) | 1,777 (31.5) | 27,742 (34.6) | 1,715 (27.8) |
| Higher | 22,754 (29.7) | 2,527 (44.7) | 23,789 (29.6) | 3,086 (50.1) |
| **Lifestyle** |  |  |  |  |
| Body fat (kg), mean (SD) | 26.1 (9.3) | 36.3 (13.5) | 22.0 (7.8) | 28.2 (10.9) |
| Total PA (MET/h/week), mean (SD) | 2,745.2 (2,835.3) | 1,882.7 (2,261.7) | 3,358.2 (3,721.4) | 2,301.5 (2,845.5) |
| Total Sedentary behaviour (h/day), mean (SD) | 4.6 (1.9) | 5.4 (2.5) | 5.4 (2.4) | 6.2 (2.9) |
| Red meat (portion.week-1), mean (SD) | 2.0 (1.3) | 2.1 (1.4) | 2.2 (1.5) | 2.4 (1.7) |
| Processed meat intake (portion.week-1), mean (SD) | 1.6 (1.0) | 1.8 (1.1) | 2.2 (1.0) | 2.4 (1.1) |
| Protein (g/day), mean (SD) | 78.6 (23.1) | 79.1 (27.2) | 87.7 (27.4) | 85.9 (32.3) |
| Calcium (mg/day), mean (SD) | 958.5 (371.2) | 953.5 (432.6) | 1,029.8 (402.8) | 1,025.6 (468.2) |
| Alcohol frequency intake, n (%) |  |  |  |  |
| Daily or almost daily | 12,780 (16.7) | 616 (10.9) | 20,590 (25.7) | 1,277 (20.7) |
| 3-4 times a week | 17,079 (22.3) | 699 (12.4) | 22,411 (28.0) | 1,158 (18.8) |
| Once or twice a week | 21,237 (27.7) | 1,260 (22.3) | 21,605 (26.9) | 1,603 (26.0) |
| 1-3 times a month | 10,231 (13.3) | 795 (14.0) | 7,087 (8.8) | 604 (9.8) |
| Special occasions only | 9,997 (13.0) | 1,314 (23.3) | 4,890 (6.1) | 781 (12.7) |
| Never | 5,351 (7.0) | 964 (17.1) | 3,616 (4.5) | 737 (12.0) |
| Smoking status n (%) |  |  |  |  |
| Never | 45,981 (60.0) | 2,800 (49.6) | 40,337 (50.3) | 1,948 (31.6) |
| Previous | 24,391 (31.8) | 1,974 (35.0) | 30,895 (38.5) | 2,691 (48.1) |
| Current | 6,303 (8.2) | 874 (15.4) | 8,967 (11.2) | 1,251 (20.3) |
| **Health status** |  |  |  |  |
| Multimorbidity, n (%) |  |  |  |  |
| 0 | 29,313 (38.2) | 642 (11.4) | 29,378 (36.6) | 627 (10.2) |
| ≥1 | 47,362 (61.8) | 5,006 (88.6) | 50,821 (63.4) | 5,533 (89.8) |
| Vitamin D (nmol/l), mean (SD) | 48.9 (20.6) | 41.9 (20.0) | 49.5 (21.0) | 44.1 (21.1) |
| Using H2 blockers, n (%) | 1,242 (1.6) | 204 (3.6) | 1,359 (1.7) | 251 (4.1) |
| Using steroid, n (%) | 647 (0.8) | 196 (3.5) | 798 (1.0) | 186 (3.0) |
| Fractures in the last five years, n (%) | 7,017 (9.2) | 736 (13.1) | 6,551 (8.2) | 658 (10.8) |
| Falls in the last year, n (%) |  |  |  |  |
| No falls | 60,931 (79.6) | 3,361 (59.7) | 69,080 (86.2) | 3,897 (63.6) |
| Only one fall | 11,476 (15.0) | 1,075 (19.1) | 7,769 (9.7) | 863 (14.1) |
| More than one fall | 4,184 (5.4) | 1,190 (21.2) | 3,242 (4.1) | 1,367 (22.3) |
| Hypogonadism, n (%) | - | - | 356 (0.4) | 41 (0.7) |
| Menopause, n (%) | 53,575 (69.9) | 4,686 (83.0) | - | - |

n: number; PA: physical activity; MET: metabolic-equivalent; SD: standard deviation. *Sarcopenia includes those with sarcopenia or severe sarcopenia.

**Table S2.** **Baseline characteristics by categories of grip strength and sex**

|  | **Women** | | **Men** | |
| --- | --- | --- | --- | --- |
|  | Normal range | Low grip strength | Normal range | Low grip strength |
| **Socio-demographics** |  |  |  |  |
| Total n, (%) | 74,949 (91.0) | 7,374 (9.0) | 81,009 (93.8) | 5,350 (6.2) |
| Age (years), mean (SD) | 55.7 (8.1) | 60.0 (6.9) | 56.7 (8.1) | 60.2 (7.2) |
| Deprivation, n (%) |  |  |  |  |
| Lower | 26,208 (35.0) | 2,026 (27.5) | 28,709 (35.4) | 1,318 (24.6) |
| Middle | 26,204 (35.0) | 2,604 (35.3) | 27,793 (34.3) | 1,664 (31.1) |
| Higher | 22,537 (30.0) | 2,744 (37.2) | 24,507 (30.3) | 2,368 (44.3) |
| **Lifestyle** |  |  |  |  |
| Body fat (kg), mean (SD) | 26.7 (9.9) | 28.2 (10.6) | 22.4 (8.2) | 23.6 (9.0) |
| Total PA (MET/h/week), mean (SD) | 2,713.6 (2,817.8) | 2,584.0 (2,794.3) | 3,331.3 (3,705.2) | 2,813.1 (3,345.0) |
| Total Sedentary behaviour (h/day), mean (SD) | 4.6 (2.0) | 4.8 (2.1) | 5.6 (2.4) | 5.6 (2.6) |
| Red meat (portion.week-1), mean (SD) | 2.0 (1.3) | 2.0 (1.3) | 2.3 (1.5) | 2.3 (1.6) |
| Processed meat intake (portion.week-1), mean (SD) | 1.6 (1.0) | 1.7 (1.0) | 2.2 (1.0) | 2.3 (1.1) |
| Protein (g/day), mean (SD) | 78.7 (23.2) | 77.7 (23.9) | 87.8 (27.7) | 84.9 (27.5) |
| Calcium (mg/day), mean (SD) | 958 (373.1) | 949.6 (390.7) | 1,031.0 (406.2) | 1,004.7 (406.4) |
| Alcohol frequency intake, n (%) |  |  |  |  |
| Daily or almost daily | 12,386 (16.5) | 1,010 (13.7) | 20,625 (25.5) | 1,242 (23.2) |
| 3-4 times a week | 16,582 (22.1) | 1,196 (16.2) | 22,379 (27.6) | 1,190 (22.2) |
| Once or twice a week | 20,550 (27.4) | 1,947 (26.5) | 21,800 (26.9) | 1,408 (26.3) |
| 1-3 times a month | 10,112 (13.5) | 914 (12.4) | 7,206 (8.9) | 485 (9.1) |
| Special occasions only | 9,951 (12.3) | 1,360 (18.4) | 5,168 (6.4) | 503 (9.4) |
| Never | 5,368 (7.2) | 947 (12.8) | 3,831 (4.7) | 522 (9.8) |
| Smoking status n (%) |  |  |  |  |
| Never | 44,620 (59.6) | 4,161 (56.5) | 39,896 (49.3) | 2,389 (44.7) |
| Previous | 23,855 (31.8) | 2,510 (34.0) | 31,624 (39.0) | 2,232 (41.7) |
| Current | 6,474 (8.6) | 703 (9.5) | 9,489 (11.7) | 729 (13.6) |
| **Health status** |  |  |  |  |
| Multimorbidity, n (%) |  |  |  |  |
| 0 | 28,411 (37.9) | 1,544 (20.9) | 28,909 (35.7) | 1,096 (20.5) |
| ≥1 | 46,538 (62.1) | 5,830 (79.1) | 52,100 (64.3) | 4,254 (79.5) |
| Vitamin D (nmol/l), mean (SD) | 48.4 (20.6) | 48.2 (20.7) | 49.2 (21.1) | 47.3 (21.1) |
| Using H2 blockers, n (%) | 1,240 (1.6) | 206 (2.8) | 1,450 (1.8) | 160 (3.0) |
| Using steroid, n (%) | 682 (0.9) | 161 (2.2) | 852 (1.0) | 132 (2.5) |
| Fractures in the last five years, n (%) | 6,830 (9.1) | 923 (12.6) | 6,669 (8.3) | 540 (10.2) |
| Falls in the last year, n (%) |  |  |  |  |
| No falls | 59,260 (79.2) | 5,032 (68.4) | 69,125 (85.5) | 3,852 (72.2) |
| Only one fall | 11,206 (15.0) | 1,345 (18.3) | 7,896 (9.7) | 762 (14.3) |
| More than one fall | 4,397 (5.8) | 977 (13.3) | 3,890 (4.8) | 719 (13.5) |
| Hypogonadism, n (%) | - | - | 362 (0.4) | 35 (0.6) |
| Menopause, n (%) | 51,760 (69.1) | 6,501 (88.2) | - | - |

n: number; PA: physical activity; MET: metabolic-equivalent; SD: standard deviation. *Sarcopenia includes those with sarcopenia or severe sarcopenia.

**Table S3.** **Baseline characteristics by categories of muscle mass and sex**

|  | **Women** | | **Men** | |
| --- | --- | --- | --- | --- |
|  | Normal range | Low muscle mass | Normal range | Low muscle mass |
| **Socio-demographics** |  |  |  |  |
| Total n, (%) | 79,190 (96.2) | 3,133 (3.8) | 86,220 (99.8) | 139 (0.2) |
| Age (years), mean (SD) | 55.9 (8.1) | 61.4 (6.0) | 56.9 (8.1) | 60.6 (6.8) |
| Deprivation, n (%) |  |  |  |  |
| Lower | 27,103 (34.2) | 1,131 (36.1) | 29,988 (34.8) | 39 (28.1) |
| Middle | 27,685 (35.0) | 1,123 (35.8) | 29,422 (34.1) | 35 (25.2) |
| Higher | 24,402 (30.8) | 879 (28.1) | 26,810 (31.1) | 65 (46.8) |
| **Lifestyle** |  |  |  |  |
| Body fat (kg), mean (SD) | 27.0 (10.1) | 22.4 (6.6) | 22.5 (8.2) | 18.3 (7.4) |
| Total PA (MET/h/week), mean (SD) | 2,706.2 (2,821.2) | 2,617.7 (2.677.9) | 3,303.4 (3,689.2) | 2,287.0 (2,087.0) |
| Total Sedentary behaviour (h/day), mean (SD) | 4.6 (2.0) | 4.6 (1.9) | 5.5 (2.4) | 5.5 (2.8) |
| Red meat (portion.week-1), mean (SD) | 2.0 (1.3) | 2.0 (1.3) | 2.3 (1.5) | 2.2 (1.6) |
| Processed meat intake (portion.week-1), mean (SD) | 1.6 (1.0) | 1.6 (1.0) | 2.2 (1.0) | 2.2 (1.1) |
| Protein (g/day), mean (SD) | 78.7 (23.4) | 75.4 (21.1) | 87.6 (27.7) | 86.5 (31.7) |
| Calcium (mg/day), mean (SD) | 959.5 (375.0 | 922.2 (356.1) | 1,029.5 (406.0) | 1,067.3 (512.4) |
| Alcohol frequency intake, n (%) |  |  |  |  |
| Daily or almost daily | 12,785 (16.1) | 611 (19.5) | 21,816 (25.3) | 51 (36.7) |
| 3-4 times a week | 17,121 (21.6) | 657 (21.0) | 23,539 (27.3) | 30 (21.6) |
| Once or twice a week | 21,714 (27.5) | 784 (25.0) | 23,179 (26.9) | 29 (20.9) |
| 1-3 times a month | 10,638 (13.4) | 388 (12.4) | 7,682 (8.9) | 9 (6.5) |
| Special occasions only | 10,896 (13.8) | 415 (13.2) | 5,663 (6.6) | 8 (5.7) |
| Never | 6,036 (7.6) | 279 (8.9) | 4,341 (5.0) | 12 (8.6) |
| Smoking status n (%) |  |  |  |  |
| Never | 46,891 (59.2) | 1,890 (60.3) | 42,231 (49.0) | 54 (38.9) |
| Previous | 25,398 (32.1) | 967 (30.9) | 33,814 (39.2) | 42 (30.2) |
| Current | 6,901 (8.7) | 276 (8.8) | 10,175 (11.8) | 43 (30.9) |
| **Health status** |  |  |  |  |
| Multimorbidity, n (%) |  |  |  |  |
| 0 | 29,023 (36.6) | 932 (29.8) | 29,967 (34.8) | 38 (27.3) |
| ≥1 | 50,167 (63.4) | 2,201 (70.2) | 56,253 (65.2) | 101 (72.7) |
| Vitamin D (nmol/l), mean (SD) | 48.3 (20.6) | 49.8 (21.0) | 49.1 (21.1) | 41.2 (23.5) |
| Using H2 blockers, n (%) | 1,378 (1.7) | 68 (2.2) | 1,608 (1.9) | 2 (1.4) |
| Using steroid, n (%) | 792 (1.0) | 51 (1.6) | 981 (1.1) | 3 (2.2) |
| Fractures in the last five years, n (%) | 7,400 (9.4) | 353 (11.3) | 7,187 (8.4) | 22 (15.8) |
| Falls in the last year, n (%) |  |  |  |  |
| No falls | 61,905 (78.3) | 2,387 (76.3) | 72,877 (84.7) | 100 (71.9) |
| Only one fall | 11,985 (15.2) | 566 (18.1) | 8,610 (10.0) | 21 (15.1) |
| More than one fall | 5,197 (6.5) | 177 (5.6) | 4,591 (5.3) | 18 (13.0) |
| Hypogonadism, n (%) | - | - | 397 (0.5) | 0 |
| Menopause, n (%) | 55,327 (69.9) | 2,935 (93.7) | - | - |

n: number; PA: physical activity; MET: metabolic-equivalent; SD: standard deviation. *Sarcopenia includes those with sarcopenia or severe sarcopenia

**Table S4. Associations between categories of sarcopenia with incident osteoporosis by sex**

| **Women** | | | | | | | |
| --- | --- | --- | --- | --- | --- | --- | --- |
|  | **Total n** | **Events** | **Normal** | **Pre-sarcopenia (low grip)** | | **Sarcopenia*** | |
|  |  |  |  | **HR (95% CI)** | **p-value** | **HR (95% CI)** | **p-value** |
| Model 1 | 74,293 | 4,321 | 1.00 (Ref.) | 1.04 (0.94; 1.16) | 0.420 | 2.01 (1.61; 2.51) | <0.001 |
| Model 2 | 74,293 | 4,321 | 1.00 (Ref.) | 1.02 (0.92; 1.13) | 0.717 | 1.70 (1.36; 2.12) | <0.001 |
| Model 3 | 74,293 | 4,321 | 1.00 (Ref.) | 1.00 (0.90; 1.11) | 0.956 | 1.66 (1.33; 2.08) | <0.001 |
| Model 4 | 73,167 | 3,195 | 1.00 (Ref.) | 1.02 (0.90; 1.15) | 0.764 | 1.79 (1.38; 2.33) | <0.001 |
| Model 5 | 32,686 | 1,618 | 1.00 (Ref.) | 1.01 (0.85; 1.21) | 0.889 | 1.48 (0.96; 2.25) | 0.072 |
| **Men** | | | | | | | |
|  | **Total n** | **Events** | **Normal** | **Pre-sarcopenia (low grip)** | | **Sarcopenia*** | |
|  |  |  |  | **HR (95% CI)** | **p-value** | **HR (95% CI)** | **p-value** |
| Model 1 | 80,136 | 1,059 | 1.00 (Ref.) | 1.40 (1.12; 1.76) | 0.004 | 4.97 (1.60; 15.4) | 0.006 |
| Model 2 | 80,136 | 1,059 | 1.00 (Ref.) | 1.34 (1.07; 1.68) | 0.012 | 3.65 (1.17; 11.4) | 0.026 |
| Model 3 | 80,136 | 1,059 | 1.00 (Ref.) | 1.30 (1.03; 1.63) | 0.025 | 3.04 (0.97; 9.54) | 0.057 |
| Model 4 | 79,890 | 813 | 1.00 (Ref.) | 1.37 (1.06; 1.78) | 0.016 | 1.53 (0.21; 11.0) | 0.671 |
| Model 5 | 34,462 | 392 | 1.00 (Ref.) | 1.37 (0.92; 2.05) | 0.120 | 6.95 (0.97; 49.8) | 0.054 |

Analyses are presented as HR with their respectively CI. Non-sarcopenic participants were used as the reference group. Analyses were adjusted by: model 1, adjusted by socio-demographic factors (age and deprivation); model 2 as model 1, but additionally morbidity count, physical activity, smoking, alcohol and red and processed meat intake. Model 3, as model 2, but additionally adjusted by corticosteroids, H2 blockers, falls and fractures in the last five years and menopause in women and hypogonadism in men. Model 4, as per model 3, but using a 2-year landmark that excluded participants who experienced events within the first two years of follow-up; and model 5, as per model 3, but further adjusted for calcium and protein intake. *Sarcopenia includes those with sarcopenia or severe sarcopenia.

**Table S5. Associations between categories of sarcopenia with subtypes osteoporosis incidence by sex**

| **Women** | | | | | | | |
| --- | --- | --- | --- | --- | --- | --- | --- |
|  | **Total n** | **Events** | **Normal** | **Pre-sarcopenia (low grip)** | | **Sarcopenia*** | |
|  |  |  |  | **HR (95% CI)** | **p-value** | **HR (95% CI)** | **p-value** |
| Pathological fracture |  |  |  |  |  |  |  |
| Model 1 | 74,293 | 1,917 | 1.00 (Ref.) | 0.91 (0.77; 1.08) | 0.278 | 1.18 (0.76; 1.83) | 0.467 |
| Model 2 | 74,293 | 1,917 | 1.00 (Ref.) | 0.90 (0.76; 1.07) | 0.249 | 1.14 (0.74; 1.78) | 0.549 |
| Model 3 | 74,293 | 1,917 | 1.00 (Ref.) | 0.90 (0.76; 1.06) | 0.217 | 1.14 (0.73; 1.78) | 0.552 |
| Model 4 | 73,858 | 1,482 | 1.00 (Ref.) | 0.92 (0.76; 1.12) | 0.423 | 1.17 (0.70; 1.95) | 0.551 |
| Model 5 | 32,686 | 643 | 1.00 (Ref.) | 1.00 (0.74; 1.35) | 0.987 | 1.29 (0.58; 2.90) | 0.532 |
| No pathological fracture/ classified elsewhere |  |  |  |  |  |  |  |
| Model 1 | 74,293 | 2,404 | 1.00 (Ref.) | 1.23 (1.07; 1.40) | 0.003 | 2.46 (1.90; 3.18) | <0.001 |
| Model 2 | 74,293 | 2,404 | 1.00 (Ref.) | 1.19 (1.04; 1.36) | 0.011 | 1.83 (1.41; 2.38) | <0.001 |
| Model 3 | 74,293 | 2,404 | 1.00 (Ref.) | 1.15 (1.01; 1.32) | 0.039 | 1.76 (1.36; 2.28) | <0.001 |
| Model 4 | 73,845 | 1,956 | 1.00 (Ref.) | 1.14 (0.98; 1.33) | 0.079 | 1.87 (1.40; 2.49) | <0.001 |
| Model 5 | 32,686 | 975 | 1.00 (Ref.) | 1.10 (0.88; 1.37) | 0.401 | 1.66 (1.01; 2.73) | 0.046 |
| **Men** | | | | | | | |
|  | **Total n** | **Events** | **Normal** | **Pre-sarcopenia (low grip)** | | **Sarcopenia*** | |
|  |  |  |  | **HR (95% CI)** | **p-value** | **HR (95% CI)** | **p-value** |
| Pathological fracture |  |  |  |  |  |  |  |
| Model 1 | 80,136 | 658 | 1.00 (Ref.) | 1.23 (0.90; 1.68) | 0.197 | 2.64 (0.37; 18.8) | 0.332 |
| Model 2 | 80,136 | 658 | 1.00 (Ref.) | 1.17 (0.86; 1.60) | 0.314 | 2.29 (0.32; 16.4) | 0.410 |
| Model 3 | 80,136 | 658 | 1.00 (Ref.) | 1.15 (0.84; 1.57) | 0.386 | 2.04 (0.28; 14.7) | 0.477 |
| Model 4 | 79,983 | 505 | 1.00 (Ref.) | 1.19 (0.84; 1.71) | 0.331 | - | - |
| Model 5 | 34,462 | 241 | 1.00 (Ref.) | 1.29 (0.75; 2.23) | 0.356 | - | - |
| No pathological fracture/ classified elsewhere |  |  |  |  |  |  |  |
| Model 1 | 80,136 | 401 | 1.00 (Ref.) | 1.68 (1.20; 2.35) | 0.002 | 8.02 (1.99; 32.3) | 0.003 |
| Model 2 | 80,136 | 401 | 1.00 (Ref.) | 1.62 (1.16; 2.26) | 0.005 | 4.38 (1.07; 18.0) | 0.040 |
| Model 3 | 80,136 | 401 | 1.00 (Ref.) | 1.48 (1.06; 2.07) | 0.023 | 3.49 (0.84; 14.4) | 0.084 |
| Model 4 | 80,073 | 338 | 1.00 (Ref.) | 1.68 (1.18; 2.40) | 0.004 | 4.68 (1.12; 19.5) | 0.034 |
| Model 5 | 34,462 | 151 | 1.00 (Ref.) | 1.49 (0.82; 2.71) | 0.192 | 14.8 (1.92; 114.1) | 0.010 |

Analyses are presented as HR with their respectively CI. Non-sarcopenic participants were used as the reference group. Analyses were adjusted by model 1, adjusted by socio-demographic factors (age and deprivation); model 2 as model 1, but additionally morbidity count, physical activity, smoking, body fat, alcohol and red and processed meat intake. Model 3, as model 2, but additionally adjusted by serum vitamin D levels, corticosteroids, H2 blockers, falls and fractures in the last five years and menopause in women and hypogonadism in men. Model 4, as per model 3, but using a 2-year landmark that excluded participants who experienced events within the first two years of follow-up; and model 5, as per model 3, but further adjusted for calcium and protein intake. -: not shown for lack of statistical power. *Sarcopenia includes those with sarcopenia or severe sarcopenia.

**Table S6. Associations between categories of sarcopenia and incident osteoporosis by age groups and sex**

|  | **Total n** | **death-**  **events** | **Normal** | **Pre-sarcopenia (low grip)** | | **Sarcopenia *** | |
| --- | --- | --- | --- | --- | --- | --- | --- |
|  |  |  | **HR (95%CI)** | **HR (95% CI)** | **p-value** | **HR (95% CI)** | **p-value** |
| **≥ and < 45 years** | | | | | | | |
| **Women** |  |  |  |  |  |  |  |
| ≥45 years | 65,436 | 4,203 | 1.00 (Ref.) | 1.06 (0.96; 1.18) | 0.259 | 1.87 (1.50; 2.34) | <0.001 |
| < 45 years | 8,857 | 118 | 1.00 (Ref.) | 2.09 (0.91; 4.83) | 0.084 | - | - |
| p-interaction |  |  |  |  | 0.093 |  | - |
| **Men** |  |  |  |  |  |  |  |
| ≥45 years | 71,871 | 1,003 | 1.00 (Ref.) | 1.27 (1.01; 1.61) | 0.039 | 3.06 (0.97; 9.62) | 0.056 |
| < 45 years | 8,265 | 56 | 1.00 (Ref.) | 2.26 (0.69; 7.41) | 0.179 | - | - |
| p-interaction |  |  |  |  | 0.433 |  |  |
| **≥ and < 55 years** | | | | | | | |
| **Women** |  |  |  |  |  |  |  |
| ≥55 years | 43,134 | 3,408 | 1.00 (Ref.) | 1.05 (0.94; 1.18) | 0.352 | 1.72 (1.36; 2.17) | <0.001 |
| < 55 years | 31,159 | 913 | 1.00 (Ref.) | 0.96 (0.69; 1.33) | 0.804 | 2.64 (1.22; 5.69) | 0.013 |
| p-interaction |  |  |  |  | 0.758 |  | 0.161 |
| **Men** |  |  |  |  |  |  |  |
| ≥ 55 years | 49,917 | 800 | 1.00 (Ref.) | 1.30 (1.01; 1.66) | 0.041 | 3.34 (1.06; 10.5) | 0.040 |
| < 55 years | 30,219 | 259 | 1.00 (Ref.) | 1.37 (0.77; 2.46) | 0.286 | - | - |
| p-interaction |  |  |  |  | 0.850 |  | - |
| **≥ and < 60 years** | | | | | | | |
| **Women** |  |  |  |  |  |  |  |
| ≥60 years | 29,351 | 2,479 | 1.00 (Ref.) | 0.98 (0.86; 1.11) | 0.702 | 1.75 (1.36; 2.24) | <0.001 |
| < 60 years | 44,942 | 1,842 | 1.00 (Ref.) | 1.17 (0.97; 1.42) | 0.102 | 1.67 (0.98; 2.84) | 0.058 |
| p-interaction |  |  |  |  | 0.098 |  | 0.908 |
| **Men** |  |  |  |  |  |  |  |
| ≥60 years | 35,654 | 609 | 1.00 (Ref.) | 1.29 (0.98; 1.70) | 0.064 | 2.32 (0.57; 9.47) | 0.243 |
| < 60 years | 44,482 | 450 | 1.00 (Ref.) | 1.30 (0.85; 1.99) | 0.223 | 5.27 (0.73; 37.8) | 0.098 |
| p-interaction |  |  |  |  | 0.846 |  | 0.477 |
| **≥ and < 65 years** | | | | | | | |
| **Women** |  |  |  |  |  |  |  |
| ≥65 years | 11,861 | 1,101 | 1.00 (Ref.) | 0.97 (0.81; 1.16) | 0.746 | 1.68 (1.21; 2.33) | 0.002 |
| < 65 years | 62,432 | 3,220 | 1.00 (Ref.) | 1.08 (0.95; 1.23) | 0.255 | 1.84 (1.35; 2.49) | <0.001 |
| p-interaction |  |  |  |  | 0.222 |  | 0.546 |
| **Men** |  |  |  |  |  |  |  |
| ≥65 years | 16,074 | 306 | 1.00 (Ref.) | 1.34 (0.94; 1.89) | 0.103 | 1.99 (0.27; 14.6) | 0.499 |
| < 65 years | 64,062 | 753 | 1.00 (Ref.) | 1.26 (0.93; 1.71) | 0.140 | 3.65 (0.90; 14.8) | 0.071 |
| p-interaction |  |  |  |  | 0.930 |  | 0.700 |

Analyses are presented as HR with their respectively CI. Non-sarcopenic participants were used as the reference group. Analyses were adjusted by deprivation, morbidity count, physical activity, smoking, alcohol and red and processed meat intake, body fat, serum vitamin D levels, corticosteroids, H2 blockers, falls and fractures in the last five years and menopause in women and hypogonadism in men (model 3). -: not shown for lack of statistical power. *Sarcopenia includes those with sarcopenia or severe sarcopenia.
